# Variolation Techniques, Medical Explanations, and Dissemination in Late Imperial China

**DOI:** 10.3201/eid3210.260729

**Published:** 2026-10

**Authors:** Linyu Guan, Shenjia Gu, Yunhai Pan, Lance E. Rodewald, Chuanxi Fu

**Affiliations:** Zhejiang Chinese Medical University, Hangzhou, China (L. Guan, S. Gu, Y. Pan, C. Fu); Chinese Center for Disease Control and Prevention, Beijing, China (L.E. Rodewald)

**Keywords:** variolation, smallpox, vaccines, immunization, administration, intranasal, history of medicine, variola virus, viruses, Imperial China

## Abstract

Variolation in China has a long history, yet the method remains underrepresented internationally, having limited recognition of medical rationale and indigenous development. We reviewed medical texts and records from late Imperial China and compared variolation chronology, techniques, and explanatory frameworks used in China versus India. Variolation in China was already in practice no later than the Chenghua–Longqing period of the Ming dynasty (1465–1572 CE), earlier than that reported in other countries. Sources from China described garment/blanket, lymph, and scab-derived inoculum methods for smallpox prevention. Scab-derived inocula, intranasal administration, and repeated passage techniques reflected empirical efforts to improve variolation reliability and reduce risk. Concepts of fetal toxin and seasonal pathogenic qi made variolation intelligible within contemporaneous medicine in China. Records from China predated sources from India and contained more detailed descriptions of variolation practices, supporting indigenous development and highlighting China’s central place in the early history of prophylactic inoculation.

Smallpox caused substantial illness and death worldwide until its eradication was certified by the World Health Organization in 1980 after sustained vaccination, surveillance, and containment efforts (*1*). Natural infection generally conferred long-lasting protection among survivors; however, case-fatality rates averaged ≈30% for variola major and, typically, <1% for variola minor viruses (*2**,**3*). Before the advent of vaccinia virus–based vaccination, communities used measures such as isolation and quarantine to limit transmission. In several regions, communities also practiced variolation, which is the intentional introduction of material from smallpox lesions to induce an infection expected to be milder than naturally acquired smallpox, conferring subsequent protection. Surviving records document variolation traditions in China, South Asia, the Ottoman Empire, and the Caucasus (*4*,*5*). Among those regional traditions, variolation in China is distinguished by an early and extensive textual record.

Variolation in China was interpreted within contemporaneous medical thought that included concepts such as fetal toxin (胎毒) and seasonal pathogenic qi (时气); the correlation between those concepts and documented practice supports the view that variolation methods developed indigenously within China. Three successive inoculation methods evolved historically during variolation development, followed by a trajectory of progressive technical refinement. Together, the evidence provides insight into early human immunologic thought.

Scholars from China have long engaged in research on the origins, transference, evolution, and historical effects of variolation. Later accounts retrospectively trace variolation in China to the Tang (618–907 CE) or Song (960–1279 CE) dynasties. Qing dynasty texts recorded nasal inoculation of smallpox in the Jiangnan (江南) region during the Tang Kaiyuan (开元) era (713–741 CE) (*6*) and variolation by a Mount Emei physician for the son of a Song prime minister (*7*). Those early origin claims lack independent corroboration, whereas the transference of variolation during the mid-to-late Ming dynasty (mid-1400s to mid-1600s CE) is well substantiated. Records describe variolation practitioners in Taiping (太平) County, Anhui (安徽) Province, during the Ming Longqing (隆庆) reign (1567–1572 CE) (*8*); practitioners in Jiangxi (江西) dated to the same period (*9*,*10*). Collectively, evidence indicates that variolation was established in Jiangxi between the Chenghua (成化; 1465–1487 CE) and Longqing eras and later spread across provinces of the Yangtze Basin, including Anhui, Zhejiang (浙江), Jiangsu (江苏), and Hunan (湖南). The practice became more widespread during the Qing dynasty and received imperial support; the Kangxi (康熙) Emperor recruited skilled physicians from Jiangxi Province to deliver inoculation at court and in Mongolia (*7*,*11*). 

During the 17th and 18th Centuries, variolation practices circulated beyond East Asia through multiple pathways, and accounts noted precedents from China. Late 17th-Century envoys from Russia reported variolation practices derived from China; variolation was also documented in Circassia, Ottoman territories, North Africa, and the Americas (*12*). India is often cited as an early center for variolation, although definitive written records date mainly from the 18th century. In the early 1700s, variolation was practiced in Ottoman Turkey; transfer of methods through Silk Road routes or via Russia has been proposed, although direct documentary evidence remains limited (*13*). Lady Mary Wortley Montagu helped introduce Ottoman variolation to Britain in the 1720s, contributing to wider adoption of this practice in Europe (*14*). Edward Jenner’s familiarity with variolation formed part of the practical context for his cowpox research (https://www.jenner.ac.uk/about/edward-jenner).

The early history of variolation in Asia has received sustained scholarly attention. Although the precise date and place of its initial development remain uncertain, surviving evidence identifies both China and India as early centers of variolation (*15*), whereas the earliest detailed records currently known are from China. Much international scholarship has focused on variolation in South Asia, particularly practices in British India during the 18th and 19th Centuries. Scholars from China have long been engaged in research on the origins, transference, development, and historical effects of variolation within their country. However, that body of work has not been fully integrated into international scholarship, which has devoted comparatively less attention to the technical development and medical explanatory framework of variolation. Therefore, we examined the documented chronology, technical development, and explanatory framework of variolation in China and assessed its significance within the global history of prophylactic inoculation.

## Methods

We collected evidence from historical sources in China on inoculation methods, chronology, and regional conveyance of variolation. We also compared documentation chronology, inoculation methods, and medical explanatory frameworks for variolation in China versus India. Ethical approval and informed consent were not required for this study because it was performed exclusively by using published literature, historical texts, and archival materials and did not involve human participants, identifiable personal data, or animal experiments.

### Analysis of Historical Medical Literature

We retrieved bibliographic records of ancient traditional Chinese medicine (TCM) on variolation (种痘) or human variolation (人痘) during the Ming and Qing dynasties from the General Catalogue of Ancient Traditional Chinese Medical Classics (中国中医古籍总目) (Duxiu database, https://www.duxiu.com). We obtained full texts from the Zhonghua Yidian (中华医典) compendium of TCM classics database (https://github.com/wanghaisheng/zhognhuayidian), the Shidian Ancient Classics (识典古籍) database (https://www.shidianguji.com), and collections from several libraries. We selected the earliest available works that had sufficiently detailed descriptions of inoculation techniques, inoculum selection, and theoretical principles of variolation; 8 works met those criteria. We conducted an in-depth reading of those works and precisely elucidated the meanings of characters, phrases, and sentences therein. We then extracted and organized information on inoculation approaches, inoculum selection, cultivation protocols for a mature inoculum, preservation conditions for variolation vaccines, inoculation timing, eligible populations for inoculation, contraindications, and postinoculation care and regimen.

We searched records from medical texts spanning the Han (202 BCE–220 CE) to the Qing (1644–1912 CE) dynasty to explore the pivotal theories proposed by physicians of successive dynasties regarding the etiology, pathogenesis, prevention, and treatment of smallpox. We conducted searches in the Encyclopedia of TCM and Shidian Ancient Classics databases by using historical terms for smallpox in China, including douchuang (痘疮), luchuang (虏疮), wandouchuang (豌豆疮), dengdouchuang (登痘疮), tianchuang (天疮), and tiandou (天痘). We selected texts recording innovative theories from each historical period or marking paradigm shifts in the understanding of smallpox for inclusion; 12 works met those criteria. We arranged the works chronologically and analyzed to trace changes in medical explanations of smallpox in China. We evaluated evidence for indigenous originality of variolation in China from 2 perspectives: the completeness of its technical system and the traceability of theoretical interpretation.

### Comparative Analysis of Variolation in China and India

The relative chronology of variolation in China and India remains uncertain. We compared available historical sources from both countries to assess the earliest documented evidence of variolation.

We developed a search strategy for variolation in India (Table 1). We identified additional sources through manual screening of the reference lists of all retrieved documents. In total, we included 35 historical review articles. Access to early indigenous medical texts in India addressing smallpox prevention and treatment is highly limited. Among the accessible sources, only 1 classic Ayurvedic work, the Bhāvaprakāśa, met the inclusion criteria. We used an English translation of the original Sanskrit.

**Table 1:** Search strategy for variolation in India in study of variolation techniques, medical explanations, and dissemination in late Imperial China*

Database	Search string
PubMed	(Smallpox inoculation[Title/Abstract] OR Variolation[Title/Abstract] OR Smallpox vaccination[Title/Abstract]) AND (India[Title] OR Bengal[Title] OR South Asia[Title] OR Punjab[Title] OR Madras[Title] OR “East Indies”[Title])
Internet archive	Mediatype:texts AND date:[1700–01–01 TO 1900–12–31] AND ((Inoculation OR Inoculating OR Variolation) AND (India OR “East Indies” OR Bengal OR Bengall OR Hindostan OR Hindoostan OR Calcutta OR Madras OR Punjab))

*PubMed, https://www.ncbi.nlm.nih.gov/pubmed; Internet archive, https://archive.org

We compared historical medical texts from China with the Bhāvaprakāśa to evaluate explanations of smallpox etiology and pathogenesis and approaches to prevention and treatment. We assessed evidence relevant to the indigenous development of variolation in China and the possibility of its transfer from India.

## Results

### Variolation Techniques

The reviewed medical texts from China described 3 principal variolation methods, the smallpox garment/blanket method (dou yi fa, 痘衣法/dou bei fa, 痘被法), lymph method (dou jiang fa, 痘浆法), and scab method (dou jia fa, 痘痂法). Scab-based methods used aqueous inocula (wet, shui miao fa, 水苗法) or dry (han miao fa, 旱苗法) preparations (*8*,*16*). Healthy children <3 years of age were generally selected, although older children were sometimes inoculated.

The garment/blanket method was described as the earliest form of variolation. Healthy children were exposed to clothing or bedding used by patients with smallpox; the aim was to induce an infection that was expected to be milder and to confer subsequent protection (Figure 1, panel A). In the lymph method, a cotton plug soaked with fluid from fully mature pustules was inserted into one nostril of the recipient (Figure 1, panel B). The scab method used scabs from smallpox skin lesions as the inoculum. For the aqueous inoculum method, the scabs were ground into powder and mixed with clean cotton and a small amount of water to form oval-shaped pellets, which were inserted into 1 nostril. For the dry inoculum method, powdered scabs were blown into the nostrils through a specially designed silver or bamboo tube (Figure 1, panel C) (*17*). Regardless of the inoculation method used, pediatric smallpox donors were required to have a stable clinical disease course, mild fever, and mild pustular eruption and be free of any critical or life-threatening symptoms, as a foundational safeguard for inoculum safety. Among the approaches, the aqueous inoculum method was recommended in official medical texts and regarded as the safest technique, followed by the dry inoculum method. The lymph method was criticized because collecting the inoculum required puncturing pustules, causing substantial pain to the child from whom the material was obtained. The garment/blanket method was considered less reliable and was not recommended. Although variolation remained hazardous, historical estimates indicated that the mortality rate from that method was substantially lower than that for naturally acquired smallpox. Reported mortality rates following variolation ranged from 0.5%–2% compared with ≈30% for naturally acquired smallpox; variolation typically produced a far milder generalized rash than that seen in regular smallpox (*1*).

**Figure 1:**
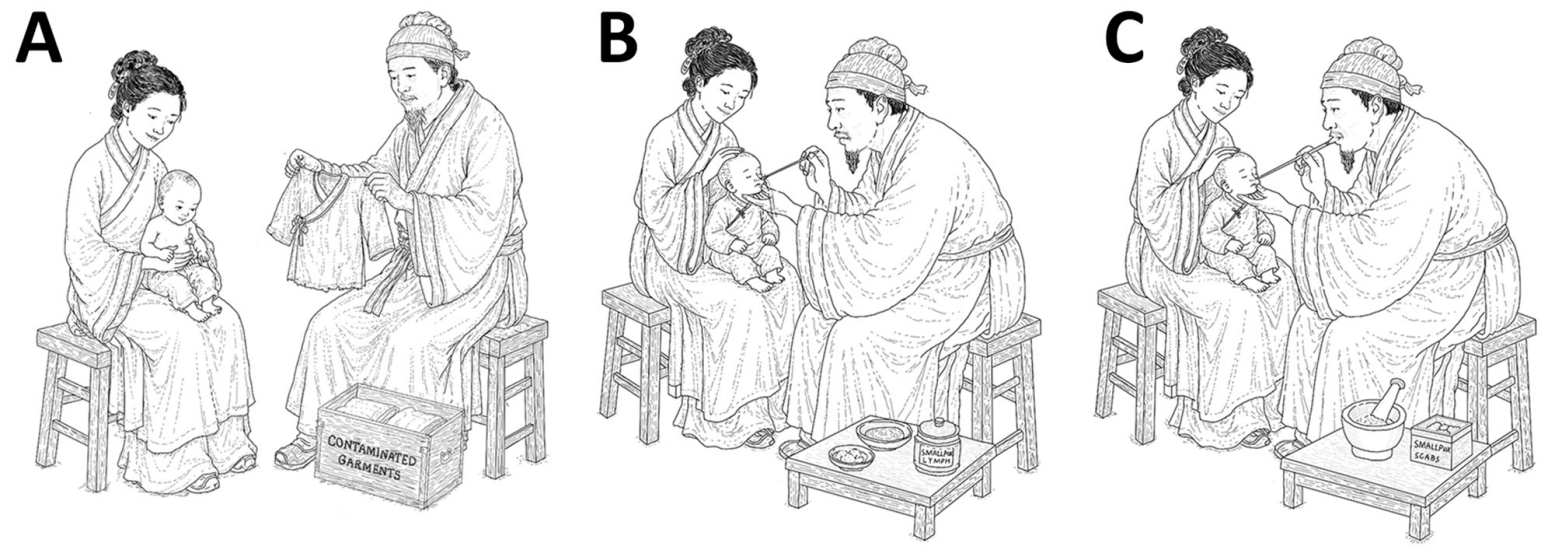
Principal variolation methods described in historical medical texts in study of variolation techniques, medical explanations, and dissemination in late Imperial China. A) Smallpox garment/blanket method, in which a healthy child was exposed to clothing or bedding used by a patient with smallpox. The aim was to induce infection that was expected to be milder and to confer subsequent protection. B) Smallpox lymph method, in which a cotton plug soaked with fluid from fully mature pustules was inserted into one nostril of the recipient. A silk thread was tied to the cotton plug to ensure that it could be safely removed. C) Smallpox scab method (dry inoculum method), in which powdered smallpox scabs were insufflated into the child’s nostril through a slender tube while the mother stabilized the child. Illustrations were hand-drawn reconstructions by an author (S.G.) according to descriptions in historical medical texts from China.

For analytical clarity, we described the documented practices using 3 modern categories without attributing modern virologic or immunologic concepts to historical practitioners. We described scab-derived inoculum, intranasal (mucosal) delivery, and serial human passage in the mature inoculum tradition.

### Scab-Derived Inoculum

Variola virus can be detected in scabs shed by convalescents; however, scabs appear to have played a limited role in virus transmission compared with respiratory spread, which was predominant in community settings (*15*). For variolation, human-derived scabs were used as the inoculum and could elicit protection by provoking a modified course of disease. After a successful infection via variolation, fever, pustulation, and scabbing typically followed; survivors generally had long-term protection. For scientific clarity, we compared variolation with vaccinia-based smallpox vaccination, the platform used in eradication campaigns, which followed standardized procedures and, in practice, often involved revaccination. Overall, scab-based variolation could confer protection but still carried risks of severe disease and secondary transmission.

### Intranasal (Mucosal) Administration

Modern evidence indicates that variola infection is initiated at the respiratory mucosa and spreads predominantly via aerosols and droplets (*18*,*19*). The nasal insufflation route of variolation in China aligned with this virus entry portal. Practitioners recognized that donors could not be screened for other infections. By avoiding percutaneous scarification, intranasal delivery might have reduced certain iatrogenic exposure pathways, although it did not eliminate infectious risks.

### Serial Human Passage in the Mature Inoculum Tradition

The reviewed texts distinguished 2 scab-based variolation traditions in China according to the inoculum source. The Huzhou (湖州; in Zhejiang Province) tradition used mild and stable scabs from naturally infected persons, known as the natural inoculum method (shi miao fa, 时苗法). The Songjiang (松江; a district previously part of the Jiangsu Province and currently a part of Shanghai) tradition used scabs derived from successive human variolations, known as the mature inoculum method (shu miao fa, 熟苗法). Variolation practitioners discovered that reactogenicity was reduced and safety increased through successive human-to-human passages. Serial passage of scab material through variolated recipients was considered to produce milder postinoculation illness and greater safety; inocula obtained after >7 passages were preferred (*20*). The mature inoculum tradition in China is best understood as an empirical approach to risk reduction through inoculum selection and repeated passage. Viewed retrospectively, the procedure resembles serial passages used in attenuation. However, historical sources neither show that practitioners understood attenuation in modern virologic terms nor document measured changes in virulence. Therefore, the mature inoculum method should not be equated with laboratory attenuation or regarded as a validated precursor of live-attenuated vaccine development.

### Indigenous Development of Variolation in China

In China, smallpox was generally regarded as an imported infectious disease, first introduced from Vietnam during the Jianwu (建武; 25–56 CE) period of Emperor Guangwu (光武; 25–56 CE) of the Eastern Han dynasty (*21*). Over subsequent centuries, physicians gradually deepened their understanding of the disease by adapting traditional medical theories to explain its etiology and pathogenesis, a process that ultimately culminated in the development of variolation. Within this intellectual environment, variolation emerged as a practice that corresponded to local theory while also corresponding to observed patterns of contagion. We emphasized compatibility between practice and explanatory framework rather than a claim of linear theory-to-practice causation. We summarized the historical development of medical knowledge of smallpox and variolation in China (Table 2) (*7*,*21*–*23*) and show a logical progression from recognition of contagion and susceptibility to preventive intervention (Figure 2). Those summaries are analytic aids generated according to historical sources and do not imply complete unanimity across texts or regions. Ancient China had a progressive theoretical logic for understanding smallpox and variolation as well as an academic context that could foster the emergence of variolation. Variolation practices in China originated from the country’s theoretical system of ancient medicine and preventive practices.

**Table 2:** Development of smallpox and variolation in study of variolation techniques, medical explanations, and dissemination in late Imperial China*

Period; key works	Development of smallpox studies in TCM	Contribution to the invention of variolation
The Wei, Jin, and Northern and Southern dynasties (3rd–6th centuries); Ge Hong’s handbook of prescriptions for emergencies (Zhouhou Beiji Fang) (*21*)	Recognized the contagiousness of smallpox (shi xing bing). Texts inferred that lesion material might participate in transmission.	Early recognition of contagion generated the idea that material from patients could induce disease in others, which was a conceptual precursor to deliberate inoculation.
Song dynasty (≈12th century); Qian Yi’s Key to Therapeutics of Children’s Diseases (Xiao’er Yao Zheng Zhi Jue) (*22*)	Proposed congenital susceptibility (pathogenic qi contracted in utero) as part of smallpox etiology. This pathogenic qi originates from the mother's behaviors during pregnancy, such as indulging in greasy and sweet foods, being frequently depressed or angry, indulging in sexual desires, or having malignant diseases, including syphilis. The toxic fire generated from those factors is stored in the essence and blood. The fetus is affected by this toxin from the mother during fetal development, leading to diseases after birth.	Laid the foundation for the etiologic theory of fetal toxin (tai du). Fetal toxin is an etiologic term in TCM.
Ming dynasty (≈15th–16th centuries); Wang Quan’s jade fragments on smallpox (Pian Yu Dou Zhen) and essential principles of smallpox (Dou Zhen Xin Fa) (*23*)	Consolidated the belief that 1 attack implied durable protection. Elucidating the etiology and pathogenesis of smallpox according to the theories of fetal toxin and external pathogens. Seasonal pathogenic qi (Shi Qi) was considered contagious, and smallpox pustules were believed to spread through interpersonal transmission. Because fetal toxin was viewed as an intrinsic bodily factor, it was believed that everyone carried the risk of developing smallpox.	Mapped historical categories to prevention, if disease confers durable protection; a milder, induced course might preempt severe natural disease, a rationale later invoked for variolation. It was also believed that a single infection could eliminate fetal toxin, rendering the person immune to future attacks. This led to the idea of inducing a mild, controlled infection through artificial means, thus, guiding the excretion of fetal toxin from the body and removing internal susceptibility and building immunity, rather than waiting for natural exposure.
Ming dynasty (≈15th–16th century); Wang Quan’s essential principles of smallpox (Dou Zhen Xin Fa) (*23*)	Identified lifelong immunity after a single smallpox infection, frequently cited later to justify deliberate, milder smallpox induction.	
Middle to late Ming to early Qing dynasties (15th–17th century); Zhu Chungu’s Conclusive theories on smallpox and rashes (Dou Zhen Ding Lun) (*7*)	Articulated “While the external factor—the pathogenic qi—was beyond human control, the internal factor, fetal toxin, could be controlled through human intervention. Before exposure to the external virus, individuals could deliberately introduce inoculum into the body to expel fetal toxin.” With its relatively low virulence, this inoculum caused only a mild smallpox infection, safely and effectively expelling fetal toxin and thus preventing life-threatening disease.	The synthesis of theory and practice provided internal coherence to variolation within TCM, enabling acceptance and widespread implementation.

*TCM, traditional Chinese medicine.

**Figure 2:**
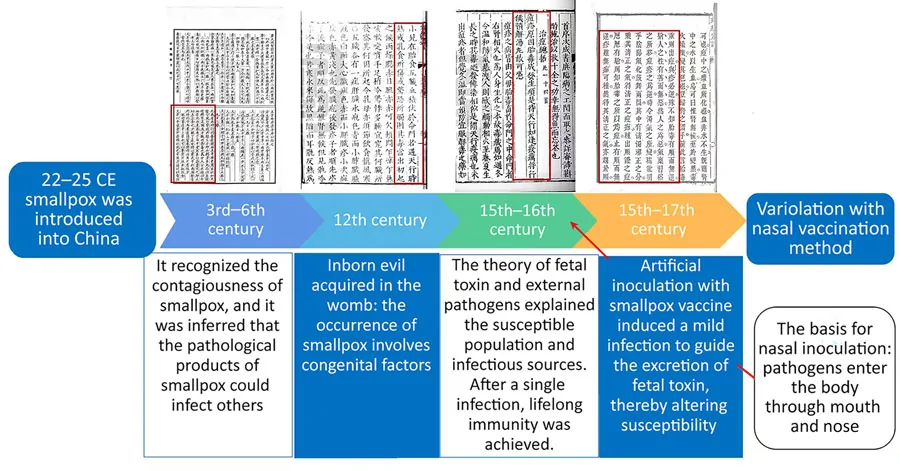
Logical progression underlying intranasal variolation in study of variolation techniques, medical explanations, and dissemination in late Imperial China. Schematic outlines the cognitive evolution of smallpox in traditional Chinese medicine that gave rise to variolation, spanning from the introduction of smallpox (25–55 CE) to recognition of its transmissibility, formulation of fetal toxin etiology, and eventual formalization of intranasal variolation. Developmental trajectory underpins the indigenous originality of variolation in China. Screenshots of ancient medical texts from China are shown at the top, sequentially sourced from references (*21*), (*22*), (*23*), and (*7*). Direct excerpts from original ancient medical text are outlined in red, serving as the primary source supporting the summary descriptions at the bottom. Schematic is only an analytical simplification and does not imply a unidirectional or deterministic progression.

### Comparative Analysis of Variolation in China Versus India

Debate over the geographic origin of variolation persists. Some authors have proposed that variolation in China was introduced from India. One account attributed to missionary physician Daniel Jerome MacGowan indicated an 11th-Century Buddhist nun in China learned the practice through a monk who had acquired it in India (*24*). However, no clear description of prophylactic variolation was identified in the Sanskrit medical material available in our study. Variolation practices were well established in India by at least the 17th–18th Centuries. The earliest written record of variolation in India dates to 1731, when Robert Coult wrote to Dr. Oliver Coult in Great Britain, describing variolation practices in the Bengal region of India (*1*). A later, detailed description of variolation in India was found in a correspondence by John Zephaniah Holwell in 1767 that was addressed to the Royal College of Physicians in London, titled An Account of the Manner of Inoculating for the Smallpox in the East Indies (*25*)*.* Variolation procedures were performed exclusively by a dedicated cohort of Brahmin practitioners. The method involved soaking sharp needles in smallpox pus, making several punctures in the depression below the deltoid muscle or on the forehead or on the fingers and wrists, and then covering the area with a small paste made from cooked rice; alternatively, the inoculation site was dressed with gauze (*26*–*29*).

We compared medical accounts from China and Ayurvedic accounts of smallpox etiology, pathogenesis, prevention, and treatment (Table 3). Ayurvedic theories on smallpox were derived from chapter 58, titled Diagnosis and Treatment of Smallpox, obtained from the English translation of Bhāvaprakāśa (*30*), one of the 3 minor classic treatises of Ayurveda. Written by Brahmin Bhāramisra of India in the 16th Century, that book records the basic theories of Ayurvedic medicine, traditional pharmaceutical knowledge in India, and the diagnosis and treatment of diseases in various disciplines.

**Table 3:** Comparison of cognitive theories on smallpox between TCM and Ayurveda in study of variolation techniques, medical explanations, and dissemination in late Imperial China*

Category	Country tradition	
	TCM, China	Ayurveda, India
Etiology	Fetal toxin and external pathogenic factors: fetal toxin is congenital and latent in the body; Shi Qi refers to pathogenic qi generated by abnormal climate, originating from nature.	Dietary disorders, including consumption of spicy, sour, penetrating, heating, burning (during digestion), dry, and alkaline foods and beverages; indigestion; overeating; sun exposure; abnormal climate. In 1767, Holwell recorded that direct cause was the mortal part of each human and animal form; indirect cause was multitudes of imperceptible animalculae floating in the atmosphere (*25*).
Pathogenesis	Shi Qi invades the body, triggers the inherent fetal toxin within, causes the fetal toxin to be expelled from inside to outside, and eventually manifests as pox eruptions on the skin.	Dietary disorders, sun exposure, and abnormal climate cause an imbalance in ≥1 of the 3 vital life energies—Vata (wind), Pitta (bile), and Kapha (phlegm)—which in turn leads to damage to the blood, muscles, and bones, as well as the formation of blisters (smallpox) on the skin, accompanied by fever.
Prevention and treatment	Treatment is divided into 6 stages: febrile, eruption, swelling, pustulation, crusting, and desquamation stages, with syndrome differentiation and treatment applied respectively. For each stage, the main focus is on differentiating the conditions of qi and blood (deficiency or excess), cold, heat, and toxic fire; corresponding prescriptions are used for replenishing qi and blood, warming the middle and dispelling cold, clearing heat and detoxifying, supporting the interior, and expelling toxins. TCM treatment of smallpox emphasizes protecting and replenishing qi and blood, believing that fetal toxin relies on qi and blood for expulsion. Prevention is variolation.	Treatment involves first fasting and inducing emesis with appropriate use of purgation. Then, according to the type of smallpox (i.e., which one or more of the vital energies are imbalanced), corresponding herbal decoctions are administered, and topical medications are applied to the skin to relieve blisters, burning sensations, and pain. The medications used are traditional natural medicines used in India. Prevention is variolation.
Inoculum	Smallpox scab (serial passaging, mature inoculum)	Smallpox pus
Inoculation site	Intranasal (mucosal)	Skin on the forearms, fingers, or forehead
Inoculation procedure	The technique evolved sequentially from the smallpox garment/blanket method to the lymph method, and the scab method was ultimately recognized as the optimal protocol.	The core operation consisted of skin puncture followed by inoculation with smallpox pus.

*Shi Qi, seasonal pathogenic qi; TCM, traditional Chinese medicine.

Reviewed medical texts from China explained that smallpox occurred when external pathogenic factors activated a latent fetal toxin within the child’s body. According to TCM, fetal toxin is a latent pathogenic factor acquired in the womb. Within this framework, variolation was understood to induce a milder episode that expelled the toxin, thereby reducing susceptibility to subsequent smallpox. The mechanism of variolation is consistent with TCM theories on the etiology, pathogenesis, and treatment of smallpox.

As reflected in the English translation of the Bhāvaprakāśa, the Ayurvedic conceptualization of smallpox does not address intrinsic bodily factors or demonstrate recognition of the body’s susceptibility. External pathogenic factors are framed as accidental exposures, including dietary disturbances, sun exposure, and climatic aberrations, which constitute a theoretical framework that offers no clear rationale for proactively modifying such factors to achieve long-term preventive effects. Within that documented medical context, the endogenous emergence of variolation within the Ayurvedic tradition appears unlikely on the basis of currently available evidence.

Taken together, we believe that it is unlikely that variolation in China was introduced from India. Instead, variolation more likely emerged within the cognitive framework of ancient TCM and has its origins in China.

## Discussion

We reconstructed the technical system and evolution of variolation in late Imperial China and identified efforts used to improve reliability and reduce health risk. The mature inoculum (shu miao) tradition might be interpreted as empirical risk reduction through inoculum selection and repeated passage. The distinctive intranasal route, aligned with variola virus’s natural respiratory entry site, raises testable questions for modern vaccinology regarding mucosal immunogenicity, reactogenicity, and the potential of dry scab-based formulations for thermostable vaccine development. The predominance of nasal insufflation over cutaneous puncture in China is plausibly linked to the Confucian tradition of filial piety, which prohibits intentional bodily injury according to classical precepts.

We evaluated and clarified the relationship between variolation and its indigenous explanatory framework or rationale in China. The TCM concept of fetal toxins provided a rationale for intentional inoculation as a preventive intervention rather than merely inducing infection. Because of the absence of a similar preventive framework in contemporary Ayurvedic medicine, indigenous emergence of variolation in China is likely, although we remain cautious about single-origin claims and acknowledge possible parallel developments elsewhere. Nevertheless, according to the earliest surviving detailed documentation, China is the country with the earliest known development and practice of variolation; variolation practitioners in China also contributed substantially to technical refinement and health risk reduction.

The transregional circulation of variolation reshapes the understanding of prevaccine medical history. As a critical node in the global network of prophylactic practices, our findings challenge Western-centric narratives by demonstrating that preventive medicine evolved via multidirectional exchange rather than a unilinear trajectory. Furthermore, Edward Jenner’s cowpox research later arose in a medical world already familiar with variolation.

The first limitation of our study is that the earliest origins of variolation in China remain uncertain because corroborating pre–Ming dynasty evidence is scarce. Second, regional variations in techniques and interpretations are not fully addressed. Translational parallels drawn here require further validation through interdisciplinary historical and experimental research. Finally, the Ayurvedic comparison relied mainly on an English translation of the Bhāvaprakāśa. Untranslated Sanskrit manuscripts, regional-language texts, and local records were not included, limiting generalization to the wider Ayurvedic variolation tradition.

In conclusion, variolation in late Imperial China developed as a coherent preventive tradition and as an empirical practice for treating smallpox. Historical medical writings from China document a progressively elaborated knowledge of smallpox pathogenesis and rationale for variolation. Together, the surviving evidence supports the view that China was the earliest country known to have developed and practiced variolation and to have documented corresponding medical explanations. The core technical features of variolation in China, including scab-derived inoculum, intranasal administration, and repeated passage in the mature inoculum tradition, reflect sustained empirical efforts to improve reliability and reduce risk. Although those practices arose in a very different historical and scientific context, they offer informed ideas for vaccine development and inoculation, particularly regarding inoculum selection, mucosal delivery, serial-passage strategies, preservation of biologic materials, and vaccination practices in public health.
